# Binned Data Provide Better Imputation of Missing Time Series Data from Wearables

**DOI:** 10.3390/s23031454

**Published:** 2023-01-28

**Authors:** Shweta Chakrabarti, Nupur Biswas, Khushi Karnani, Vijay Padul, Lawrence D. Jones, Santosh Kesari, Shashaanka Ashili

**Affiliations:** 1 Rhenix Lifesciences, Hyderabad 500038, India; 2 Department of BioSciences and BioEngineering, Indian Institute of Technology, Guwahati 781039, India; 3 CureScience, 5820 Oberlin Dr, 202, San Diego, CA 92121, USA; 4 Department of Translational Neurosciences, Pacific Neuroscience Institute and Saint John’s Cancer Institute at Providence Saint John’s Health Center, Santa Monica, CA 90404, USA

**Keywords:** imputation, missing data, time-series data, binning, wearables

## Abstract

The presence of missing values in a time-series dataset is a very common and well-known problem. Various statistical and machine learning methods have been developed to overcome this problem, with the aim of filling in the missing values in the data. However, the performances of these methods vary widely, showing a high dependence on the type of data and correlations within the data. In our study, we performed some of the well-known imputation methods, such as expectation maximization, k-nearest neighbor, iterative imputer, random forest, and simple imputer, to impute missing data obtained from smart, wearable health trackers. In this manuscript, we proposed the use of data binning for imputation. We showed that the use of data binned around the missing time interval provides a better imputation than the use of a whole dataset. Imputation was performed for 15 min and 1 h of continuous missing data. We used a dataset with different bin sizes, such as 15 min, 30 min, 45 min, and 1 h, and we carried out evaluations using root mean square error (RMSE) values. We observed that the expectation maximization algorithm worked best for the use of binned data. This was followed by the simple imputer, iterative imputer, and k-nearest neighbor, whereas the random forest method had no effect on data binning during imputation. Moreover, the smallest bin sizes of 15 min and 1 h were observed to provide the lowest RMSE values for the majority of the time frames during the imputation of 15 min and 1 h of missing data, respectively. Although applicable to digital health data, we think that this method will also find applicability in other domains.

## 1. Introduction

In time-series data, there are many instances where there are no data available or they are missing. This could be due to various reasons, such as observing the data in the given time window not being possible; the failure of the system recording the data; or the data recorded being found to be noise and, hence, removed [1,2,3]. These missing values cause difficulties during any statistical analyses due to the requirement of a complete dataset. Few methods can analyze data with missing values, but, commonly and preferably, the missing values are imputed [4,5,6].

Analyses of incomplete data have led to the development of various imputation methods. There are different imputation categories based on the type of method and the output [7]. For instance, a few methods impute only a single value, neglecting the introduction of uncertainty into the data [8]. Other methods apply multiple imputations to compute the uncertainty of the imputation [9]. In such cases, calculations of the standard error from the variability of estimates and even confidence intervals are possible, e.g., in Monte Carlo-based methods [10]. Missing data can be present in different ways: they can be missing at random (MAR), they can be missing completely at random (MCAR), and they cannot be missing at random (MNAR) [11]. The MCAR mechanism is restrictive due to the assumption that missing data are a random sample extracted from the observed values. Hence, assuming MAR can be more realistic [12]. The imputation is also dependent on the type of data.

Wearable healthcare devices are gaining popularity due to their real-time monitoring of physical parameters, such as heart rate, step count, and calories burned, in a cost-effective way [13]. However, these primary data have missing points, which hinder downstream analyses and may provide misleading information, while correlating primary data with disease conditions [3,14]. To overcome the limitation of incomplete datasets, different imputation methods are used to fill in the missing points. In this work, we focused on the expectation maximization (EM) algorithm, k-nearest neighbor (kNN), iterative imputer (II), random forest (RF), and the simple imputer (SI) method to impute missing heart rate data.

The EM algorithm is one of the most used imputation methods, and it was proposed by Hartley and Hocking [15]. It is an iterative method that replaces missing values with the most likely data based on the empirical mean and the variance-covariance matrix in the data [16]. This algorithm is implemented well, and most statistical software programs perform EM imputation methods [17,18]. The II algorithm involves the substitution of each piece of missing data with any plausible value randomly drawn from the dataset [19,20]. It is an extension of the multiple imputation method [21]. To predict the missing data, a regression line is fitted on the available values of the feature. Each feature is modeled as a function of another feature [22]. The k-nearest neighbor (kNN) algorithm is an instance-based estimation procedure using the ‘k’-nearest neighbor values of the feature. The value that is imputed is generally the mean of the k-nearest neighbor values, and they can also be modified accordingly [23]. The kNN imputation method has been successfully applied in the real-time processing of data due to its simplicity and high accuracy [18,24]. The machine-learning-based RF imputation method takes care of the nonlinearity of data. The data are imputed based on the proximity values predicted by the random forest model [25]. The imputation is iterated to improve the result [26,27]. We also used the SI imputation method, which is a univariate algorithm that performs imputation on univariate data. Values are imputed with a constant value obtained using statistical methods, such as the mean, median, or mode [28]. The above-mentioned methods are global structure methods and left-censored methods that have shown robust performance in numerous previous studies [29].

However, these imputation methods might not always provide satisfactory results for reasons such as seasonality within data. Therefore, to improve the accuracy of the aforementioned imputation methods, we propose a new approach to impute the missing values. In this paper, we primarily focused on heart rate data and the number of steps data from wearables. Though such data are continuously recorded, very often, they contain a number of missing values. In spite of several reports [4,5,6,14], a robust method for the imputation of clinical time-series data is still lacking. Heart rate data are used for correlating and diagnosing several disease conditions, such as cardiovascular, neurological, and metabolic disorders [30]. Hence, the imputation of missing parts of such time-series data is important, as it could impact clinical decision-making systems [31,32]. Heart rate data also bear seasonality, cyclicality, and periodicity. This raises the question as to whether an entire dataset spanning 24 h of each day is required to train imputation models. Here, we performed an exploratory analysis to identify a robust way of imputation considering the above features of heart rate data. We validated our hypothesis that using the bins of training data provides better imputed values than the imputed values obtained using a complete dataset. We performed imputation on four 1 h and 15 min time frames, namely, 3–4 am, 3–4 pm, 3–3:15 am, and 3–3:15 pm, and to evaluate the performance of these imputation methods, we used the root mean square error (RMSE) evaluation metric. Comparative studies mostly rely on the accuracy of measure-based performances, such as RMSE, as they determine central tendencies [33,34]. We also went on to find the optimal bin size that could be preferred while performing imputation.

## 2. Materials and Methods

We considered a time series as *x(t),* where *x* is the variable recorded at time *t*. A time series with no missing data can be represented as

$$x_{t0},\;x_{t0+a},\;x_{t0+2a},\;x_{t0+3a},\;x_{t0+4a},\;x_{t0+5a},\;x_{t0+6a},\;x_{t0+7a},\;x_{t0+8a},\;x_{t0+9a},\;x_{t0+10a},\;\ldots \;\;$$

where data recording started at time *t*_0_, and data were recorded at time interval a. In this study, we focused on a time series where data are missing continuously and are represented as *Nan* (not a number) values as shown here:

$$x_{t0},\;x_{t0+a},\;x_{t0+2a},\;Nan,\;Nan,\;Nan,\;Nan,\;Nan,\;x_{t0+8a},\;x_{t0+9a},\;x_{t0+10a},\;\ldots \;\;$$

To impute the missing values, we applied the method of binning the data into different bin sizes ranging from 15 min to 6 h for different imputation methods. We then compared their accuracies and explored the impact of data binning by examining how it was useful in improving the accuracies of each imputation method. The efficiency of the binning method on the dataset was assessed by comparing the different imputation methods based on RMSE. All the computations were performed using the Python programming language on the Anaconda platform (Anaconda Software Distribution, USA, version Anaconda3-2020.07).

### 2.1. Data

The data were obtained from the wearable device Fitbit version 2 from 3 healthy individuals who volunteered in this research and 1 healthy volunteer wearing a Mi band. For each volunteer, 30 random days with no inherent missing data were selected from a pool of data covering 4 months. The data from the Fitbit device were available in 5-s intervals. After performing the data cleaning and smoothening, the second-level data were further converted into minute-level data by taking the average of the heart rates measured within each minute.

### 2.2. Missing Value Generation

**Figure 1:**
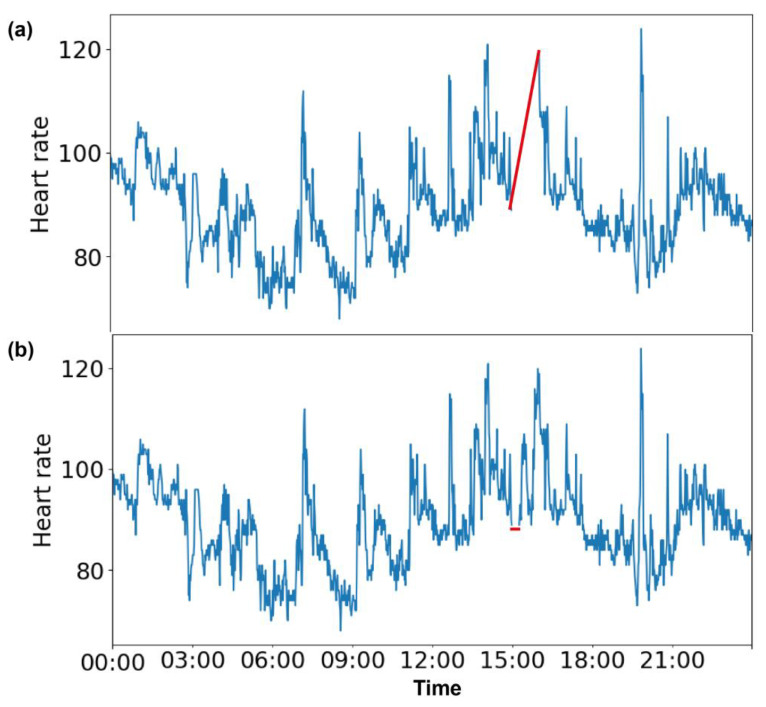
Continuous missing values in heart rate data. Data missing for 1 h from (**a**) 3 pm (15:00 h) to 4 pm (16:00 h) and (**b**) for 15 min from 3 pm (15:00 h) to 3:15 pm (15:15 h) are indicated by red lines.

To evaluate the different imputation methods, heart rate values from different time frames were deliberately removed. We worked on two time frames: one had a duration of 15 min, and the other had a longer duration of 1 h. The time frames were chosen based on the active and inactive time periods of a day. We considered 3–4 am as an ‘inactive’ period, as that was when all of the volunteers were asleep (step counts were 0). We considered the afternoon time of 3–4 pm as an ‘active’ period, as our volunteers were involved in various activities during that time. To perform an imputation analysis, we chose two sizes of missing data, 15 min and 1 h, for both the active and inactive periods. Precisely, we chose 3 to 3:15 am and 3 to 3:15 pm to perform imputation on 15 min of missing data and 3 to 4 am and 3 to 4 pm to perform imputation on 1 h of missing data. All the available heart rate values within those two time frames were replaced with *Nan* for 30 days. Figure 1a shows the missing data for 3 pm (15:00 h) to 4 pm (16:00 h), and Figure 1b shows the missing data for 3 pm (15:00 h) to 3:15 pm (15:15 h), as indicated by the red lines.

### 2.3. Data Binning

The next step was to bin the dataset into different bin sizes. The bin sizes of 15 min, 30 min, 45 min, 1 h, 2 h, 3 h, 4 h, 5 h, and 6 h were chosen, keeping the 3–4 am, 3–3:15 am, 3–4 pm, and 3–3:15 pm time frames in the center.

### 2.4. Data Imputation

Binning was followed by the performing of imputation, which was carried out using five different imputation methods, namely, EM, II, kNN, RF, and SI. Among them, SI is a simple statistical method, and the remaining are machine learning methods. We chose different types of frequently used methods for a comparative study [20]. The models were fit based on an entire month’s data and bins of the data from the volunteers. Finally, imputation was performed for the four time frames (3–4 am, 3–3:15 am, 3–4 pm, and 3–3:15 pm). EM and RF were applied using the impyute and missForest methods from the missingpy Python module, respectively. II, kNN, and SI were performed using IterativeImputer, kNNImputer, and SimpleImputer from the Scikit-learn Python module, respectively [28].

### 2.5. Performance Evaluation of Binning

This step involved evaluating the performance and robustness of the imputations and binning methods used in this study. The metric used for the evaluation of the accuracy of the estimation of the imputed values was *RMSE*, which calculates the error between the actual and the imputed values. RMSE works well as an error metric for a given variable due to its simple interpretability [35]. Considering *R_i_* and *I_i_* as the actual value and the imputed value for data point *i*, respectively, where *N* is the number of missing values, the RMSE is calculated as follows:

$$RMSE=\sqrt{\frac{\sum_{i=1}^{N}{\left(R_{i}-I_{i}\right)}^{2}}{N}}$$

## 3. Results

In this study, we examined whether the binning of a dataset improves the accuracy of various imputation methods. We used data obtained from the digital wristbands of four volunteers, covering 4 months. The device details and data acquisition details are mentioned in the Section 2. To perform the imputation, we removed consecutive data points for both 15 min and 1 h time frames. This process was repeated for 30 randomly chosen days for each volunteer.

The imputation methods used in this study were EM, II, kNN, RF, and SI. Imputation was performed for 15 min and 1 h of data, for which the following data bin sizes were used: 15 min, 30 min, 45 min, 1 h, 2 h, 3 h, 4 h, 5 h, and 6 h, as well as the whole 24 h data of 30 days, henceforth referred to as ‘total’ data.

### 3.1. Imputation of 1 h of Missing Data

**Figure 2:**
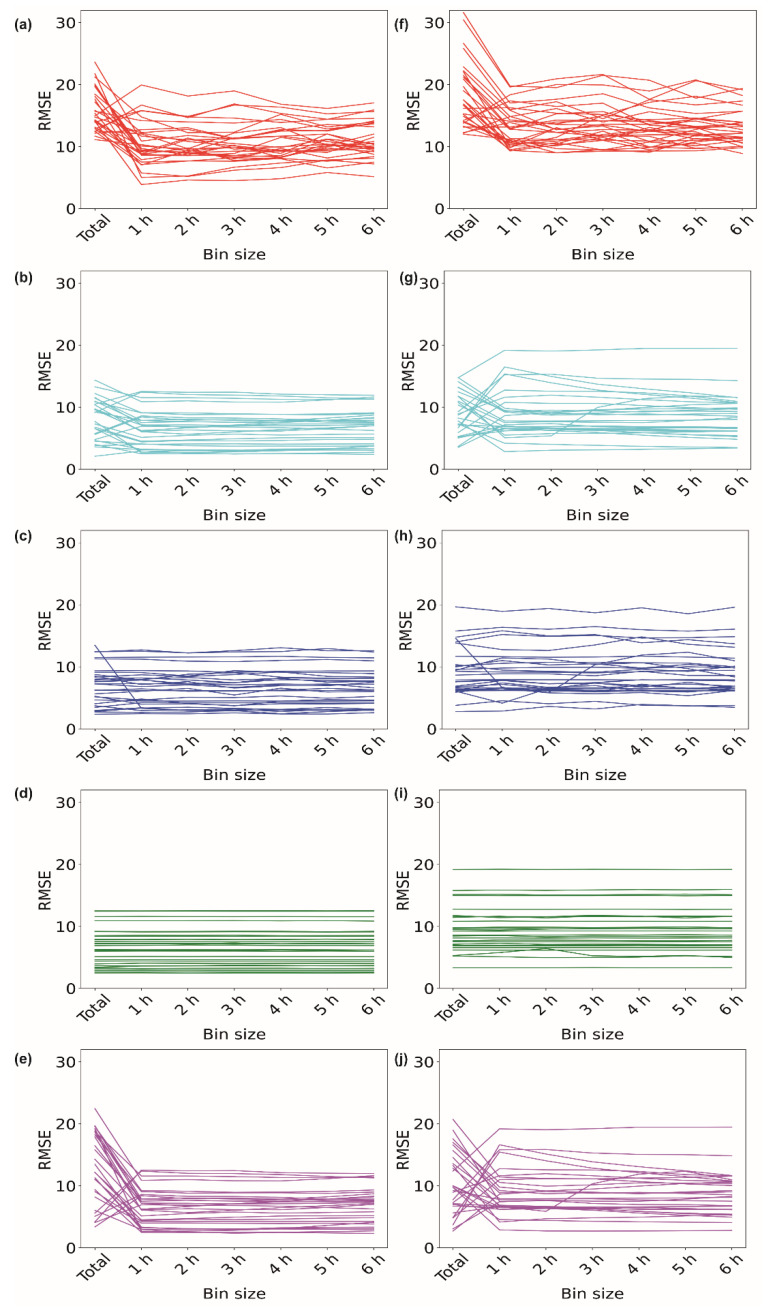
Imputation of 1 h of missing data. Variations in RMSE when data of different bin sizes were used for imputing missing data of ‘inactive’ period of 3–4 am using (**a**) EM, (**b**) II, (**c**) kNN, (**d**) RF, and (**e**) SI methods. Variations in RMSE when data of different bin sizes were used for imputing missing data of ‘active’ period of 3–4 pm using (**f**) EM, (**g**) II, (**h**) kNN, (**i**) RF, and (**j**) SI methods.

Figure 2a–e illustrate the performances of the different imputation methods for the data obtained from volunteer V1 for the 1 h missing period during the ‘inactive’ time period of 3:00–4:00 am for 30 days. For each method, the RMSE was plotted against the size of the data used for the imputation. For the EM method, the RMSE of 4 days took the highest value when the ‘total’ data were used for the purpose of imputation (Figure 2a). We observed that, when data with a 1 h bin size, i.e., data expanding from 3:00 am to 4:00 am, were used for the imputation, the RMSE dropped for 25 days, indicating a better imputation. When data with higher bin sizes were used for the imputation, the RMSE values tended to increase. This implies that data with a 1 h bin size provide better imputed values for the EM algorithm. We further explored the other algorithms. For II, out of 30 days, 21 days showed the lowest RMSE when data with a 1 h bin size were used for imputation (Figure 2b). However, for the kNN method, the RMSE dropped for only one day when data with a 1 h bin size were used (Figure 2c). The RMSE values remained almost insensitive to the data size for the RF method (Figure 2d). In the case of the SI method, for 26 days, the RMSE took a minimum value for data with a 1 h bin size. We did not observe any change in the RMSE when data with bin sizes of 2 h to 6 h were used (Figure 2e).

**Figure 3:**
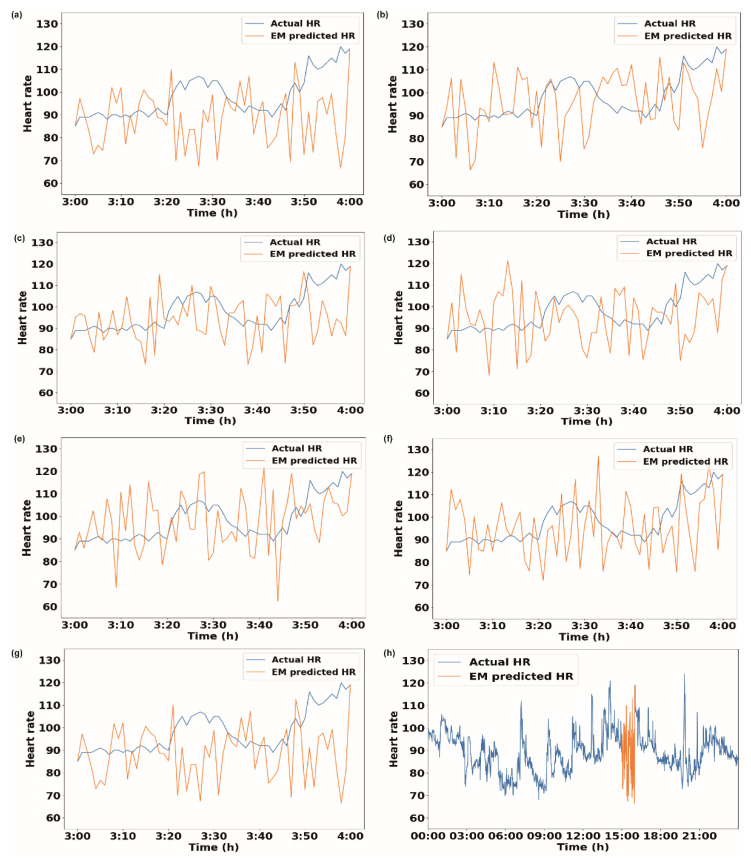
Actual heart rate data and imputed heart rate data using EM methods for 1 h of missing data from 3 pm to 4 pm. The bin sizes are (**a**) 1 h, (**b**) 2 h, (**c**) 3 h, (**d**) 4 h, (**e**) 5 h, (**f**) 6 h, and (**g**) entire data. (**h**) Heart rate data for entire day along with imputed values obtained from EM method using data with 1 h bin size when data were missing from 3 pm to 4 pm.

We further extended our analysis to the ‘active’ time period of 3:00–4:00 pm for 30 days for volunteer V1 (Figure 2f–j). Figure 2f shows the results of the EM method, where 23 days out of 30 days showed decreases in the RMSE values when data with a 1 h bin size were used for imputation. Similarly, for the II method, the RMSE reduced for 16 days when 1 h binned data were used (Figure 2g). For the ‘active’ period, the kNN method provided a lower RMSE for 5 days when binned data were used (Figure 2h). The RF method again remained insensitive for the ‘active’ period (Figure 2i). SI also maintained a similar trend for the ‘active’ period, showing a drop in the RMSE for 17 days (Figure 2j) when binned data were used compared to the ‘total’ data. Figure 3a–g show the heart rate data imputed by the EM method using data with different bin sizes. It also shows the actual heart rate data. Figure 3h shows the whole-day data, along with the data imputed by the EM method with a 1 h bin size. Supplementary Figures S1, S3 and S5 show the imputation data for the 1 h missing period for volunteers V2, V3, and V4, respectively.

### 3.2. Imputation of 15 min of Missing Data

**Figure 4:**
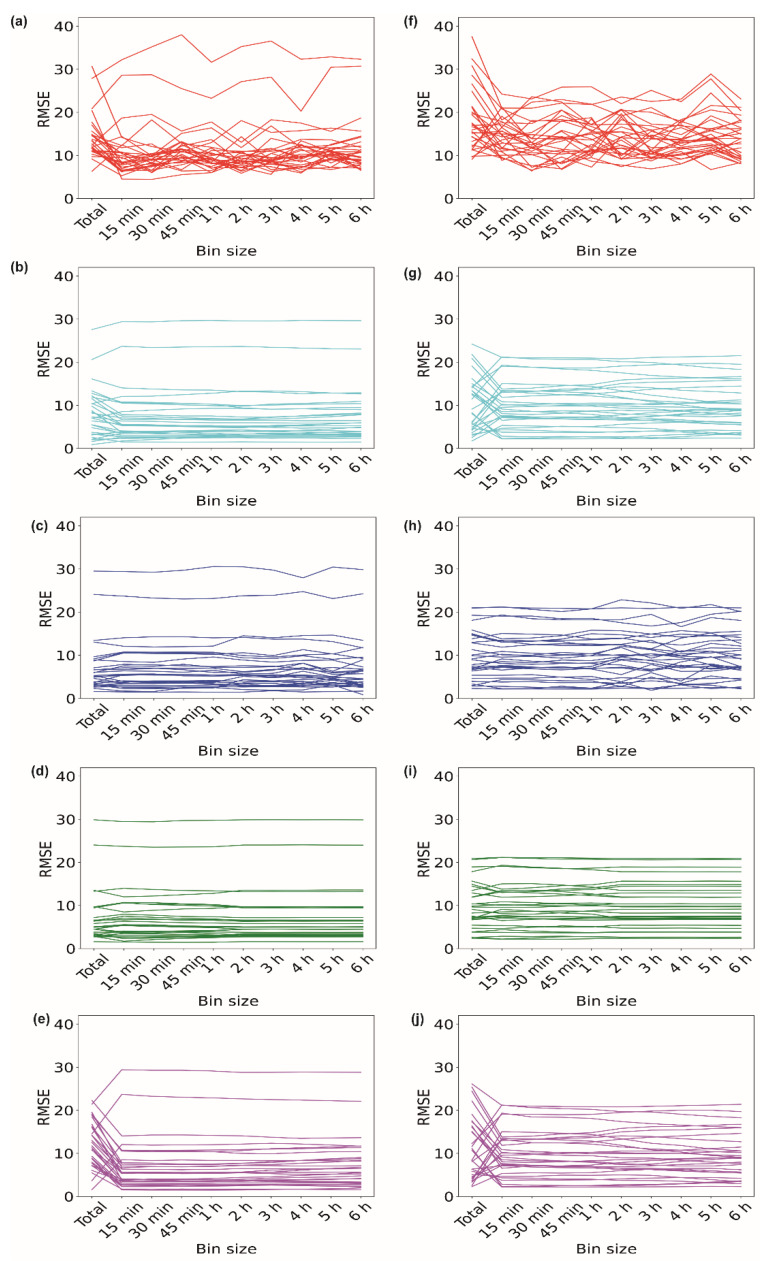
Imputation of 15 min of missing data. Variations in RMSE when data with different bin sizes were used for imputing missing data of ‘inactive’ period of 3–3:15 am using (**a**) EM, (**b**) II, (**c**) kNN, (**d**) RF, and (**e**) SI methods. Variations in RMSE when data with different bin sizes were used for imputing missing data of ‘active’ period of 3–3:15 pm using (**f**) EM, (**g**) II, (**h**) kNN, (**i**) RF, and (**j**) SI methods.

We further explored whether binned data remain effective when data are missing for a shorter time period. We conducted a similar analysis of when imputation is needed for 15 min of missing data. Figure 4a–e illustrate the performances of the different imputation methods for the data obtained from volunteer V1 for the 15 min missing period during the ‘inactive’ time period of 3:00–3:15 am. We observed that, when data with a 15 min bin size, i.e., data expanding from 3:00 am to 3:15 am, were used for imputation, the RMSE dropped for 23 days, indicating a better imputation. While exploring other algorithms, we found that, for II, 20 days showed lower RMSE values when data with a 15 min bin size were used for imputation (Figure 4b). However, for the kNN method, the RMSE dropped when data with a 15 min bin size were used for only 7 days (Figure 4c). The effect of binning was not observed when the RF imputation method was used (Figure 4d). For the SI method, the RMSE of 26 days for data with a 15 min bin size showed lower values than the ‘total’ data size. We did not observe any change in the RMSE when data with bin sizes of 2 h to 6 h were used (Figure 4e).

Similarly, the ‘active’ time period of 3:00–3:15 pm for 30 days for volunteer V1 was analyzed (Figure 4f–j). In Figure 4f, one can observe that, for the EM method, 22 days out of 30 days showed decreases in the RMSE values when data with a 1 h bin size were used for imputation. For the II method, the RMSE reduced for 15 days when data with a 15 min bin size were used (Figure 4g). In the case of the kNN method, the RMSE for only 5 out of 30 days decreased compared to that of the ‘total’ data (Figure 4h). A lower RMSE was observed in only 5 out of 30 days when the RF method was used (Figure 4i). SI also maintained a similar trend for the ‘active’ period, showing a drop in the RMSE for 17 days (Figure 4j) when binned data were used compared to the ‘total’ data. Based on these observations, we can say that the use of binned data provides a better imputation. Supplementary Figures S2, S4, and S6 show the imputation data for the 15 min missing period of volunteers V2, V3, and V4, respectively.

### 3.3. Quantitative Analysis

**Figure 5:**
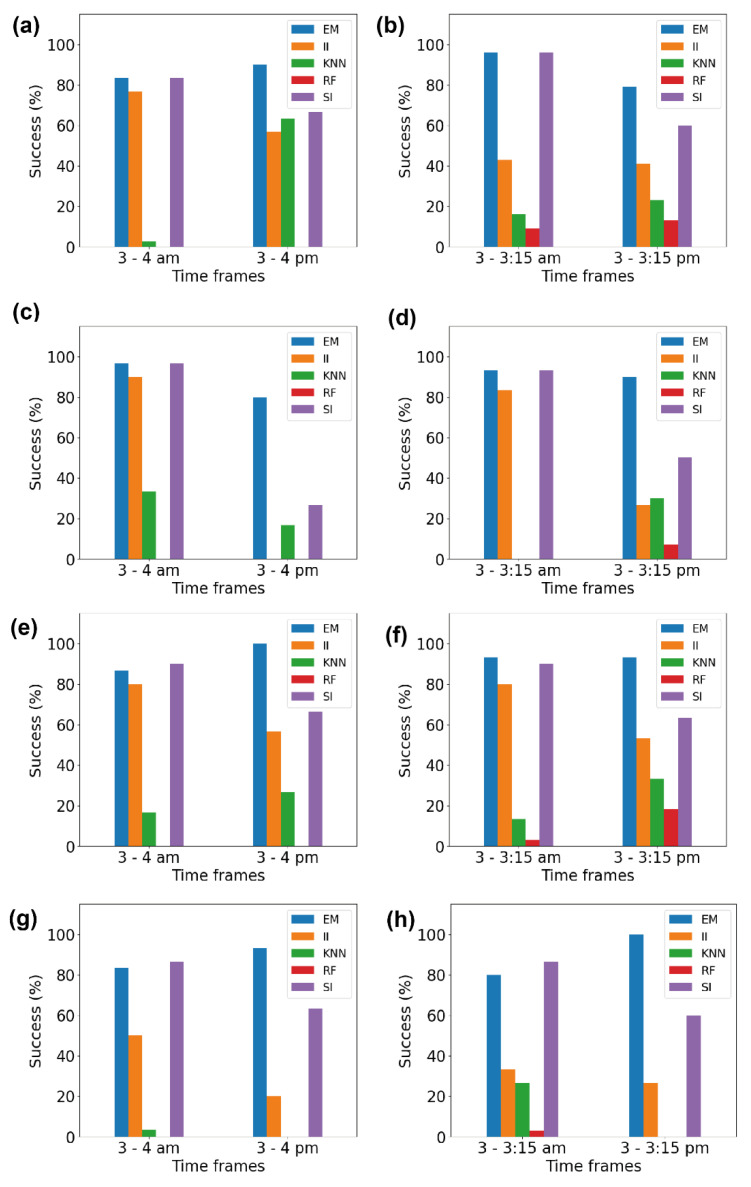
The success rates of each imputation method: (**a**,**b**) show the success rates for volunteer V1, (**c**,**d**) show the success rates for volunteer V2, (**e**,**f**) show the success rates for volunteer V3, and (**g**,**h**) show the success rates for volunteer V4.

We further tried to quantify our observations on the effect of using binned data in imputation. Figure 5 illustrates the quantitative measures of whether binned data give better RMSE values than the entire data for all volunteers. The ‘success’ rate was obtained based on the number of days showing reductions in the RMSE when binned data were used. There were some days where the binning had no effect, as the RMSE value either did not reduce or remained unchanged.

Figure 5a depicts the success rate for the 1 h imputation of data for both the ‘active’ and ‘inactive’ time periods of volunteer V1. The EM method showed a success rate above 80% in both time frames, i.e., 3–4 am and 3–4 pm. The success rate of the II method was 76% and 56% for the ‘inactive’ and ‘active’ time periods, respectively. The success rate varied hugely in the case of the kNN method, as the success rate was 2.7% during the ‘inactive’ period and 63% during the ‘active’ period. However, the RF method completely failed to show the effect of the binning. The SI method was successful, with a rate of 83% for the ‘inactive’ time period and 66% for the ‘active’ time period.

Figure 5b illustrates the success rate for the 15 min imputation of data for volunteer V1. The EM method showed a success rate of 96% for the ‘inactive’ time period and 76% for the ‘active’ time period. The success rates of the II method were 56% and 53% for the ‘inactive’ and ‘active’ time periods, respectively, quite similar in both cases. The kNN method showed a success rate of 11% during the ‘inactive’ period and 23% during the ‘active’ period. However, the RF method, again, completely failed to show the effect of the binning. The SI method was successful, with a rate of 96% in the ‘inactive’ time period and 58% in the ‘active’ time period. Figure 5c–h depict the success rates for volunteers V2, V3, and V4. We observed that, for V2, V3, and V4, at least one imputation method (the EM method) achieved a success rate of 80% or higher irrespective of the amount of missing data and the activeness of the volunteer. This high success rate validates our hypothesis that the use of binned data provides a better imputation irrespective of the amount of missing data.

### 3.4. Optimal Bin Size

**Figure 6:**
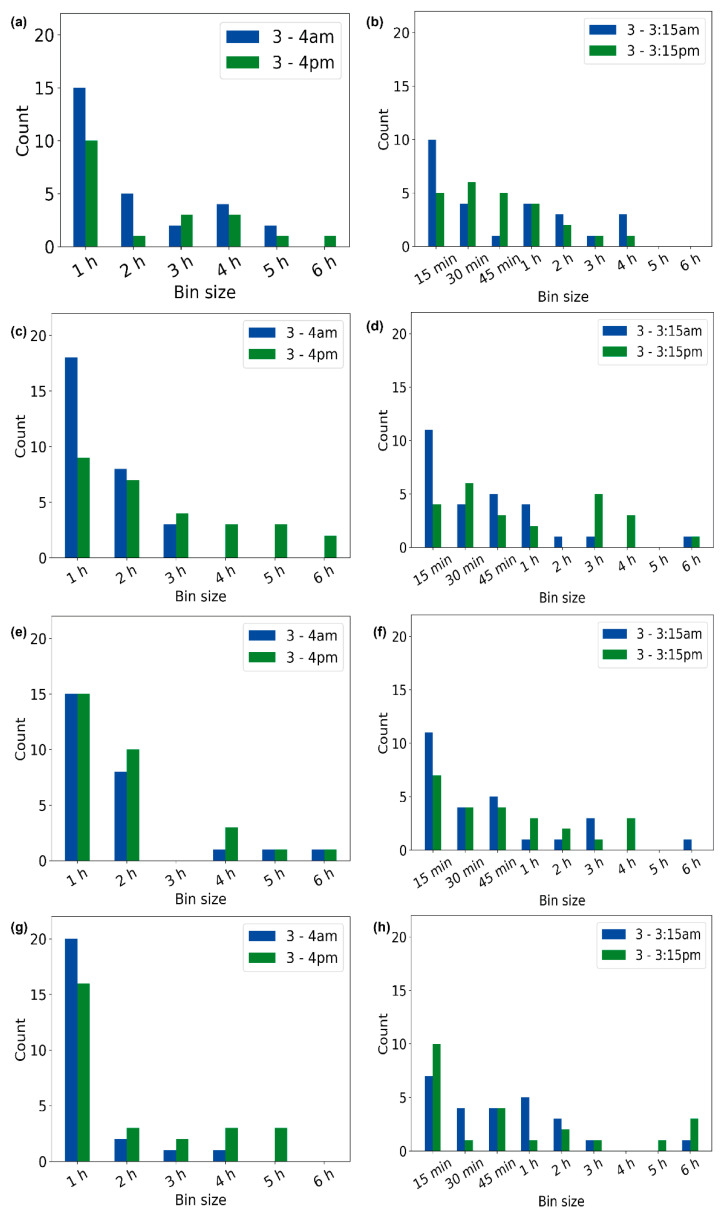
Dominance of the optimal bin size for active and inactive periods. For 1 h and 15 min of missing data for volunteers V1 (**a**,**b**), V2 (**c**,**d**), V3 (**e**,**f**), and V4 (**g**,**h**).

Next, we investigated which bin size was the most optimal. The optimal bin size could be defined as the bin size where all five imputation methods together show the minimum RMSE value. Based on the frequency of bin size resulting in the optimal bin size, we observed that, for volunteer V1, a 1 h bin size occurred for 15 days for the time frame of 3–4 am and 10 days for the time frame of 3–4 pm (Figure 6a). For the 15 min of missing data, a 15 min bin size occurred 10 times for the time frame of 3–3:15 am and 5 days for the time frame of 3–3:15 pm (Figure 6b). Figure 6c–h depict the distributions of the optimal bin sizes for volunteers V2, V3, and V4. These findings suggest that using the minimum bin size to perform imputation could result in lower RMSE values and, hence, a better imputation. These findings could also imply that choosing a bin size that is the same as the duration for which the imputation is performed could be the right decision. However, this was not true for all cases. This could be because of the insufficiency of the data for some days, which could have affected the result. The optimal bin size also varied for all dates, especially during the active period of the day (3–4 pm and 3–3:15 pm).

## 4. Discussion

We observed for most of the imputation methods that the use of data binned around the missing data provides a better imputation. The reason for this can be attributed to the inherent variation within the heart rate data. A person’s heart rate varies over the course of a day depending on their lifestyle. As lifestyle often follows some routine activities, for example, remaining physically inactive every day during the period of 3–4 am, routines are also reflected in heart rate data. Hence, to impute missing data for a certain time period of a particular day, data from other days of the same time period are adequate. Using binning for imputation could be really fruitful when handling big data, especially multivariate data. Binning makes a predictive model more effective and interpretable. Binning helps in the denoising of data, reducing the effects of outliers. However, binning could result in a power loss due to increased model parameters [36]. While proposing a new imputation method by combining the properties of missForest (MF) and local linear forest (LLF), a study by Rao et al. also showed how binning, along with careful initializing, improves imputation results to a great extent when used on multiple imputation methods [21]. Korkuć et al. performed imputation on the bins of data based on the minor allele frequency (MAF), and it was observed that bins with lower MAFs showed better imputation accuracies [37]. Another study performed imputation on patient health data, which are usually available in a few intervals or bins. It was concluded that binning and time censoring could be considered for such data where anonymity is maintained and multiple imputation methods are applied [38]. Moreover, the use of binned data means the use of a lower amount of data, which reduces computational load and data requirements. The use of binned data is applicable to any imputation algorithm. So, the choice of the algorithm is up to the researcher.

It was noted that the EM method was highly effective when data binning was used to perform imputation, as the EM method showed the highest ‘success rate’ for all the time frames and for all four volunteers. The EM method does not have any hyperparameters. It treats missing values as hidden variables and, hence, works best for MAR data. The likelihood maximization is achieved by marginalizing over the hidden variables [39]. Overall, the performance order of each method was as follows: the EM method, followed by SI, II, and kNN; RF was the only method that failed to show any effects of binning. RF-based imputation methods are not recommended for data with outcome-dependent MAR [26]. The precise reason for the insensitivity of RF in imputation using data binning is still unknown, as a very low number of studies have been conducted to date on imputation using data binning. However, we could observe that the EM method, with the highest success rate, showed a very high range of RMSE values. However, methods such as RF and kNN showed low ranges of RMSE values in either case (total data or binned data). Earlier reports have also shown that the EM method outperforms several statistical imputation methods when datasets comprise MAR-type missing data [40].

Time-series missing data are often imputed after converting the data to frequency domain, which extracts different harmonics [41]. Least square spectral and wavelet analyses are often performed for unequally spaced and non-stationary time-series data of geophysics [42,43]. This has also led to the combining of Fourier transform with other imputation algorithms for biomedical data [44]. These methods are helpful when time-series data have inherent periodicity. Splitting into harmonics means splitting into different window sizes, which considers data over the whole day. Heart rate data often bear periodicity due to the routine activity of the user. Imputation in frequency domain may be a better choice if the user performs similar activities for multiple time frames spanning different times of the day. However, the imputation can be performed considering data of similar time periods of other days irrespective of the user’s activity over the day.

From this study, we can definitely say that data binning helps in improving the RMSE values compared to the use of whole datasets for imputation. The researcher must select the optimal bin size for their dataset by either depending on the time duration for which they want to perform the imputation or by performing the various bin sizes and selecting accordingly. As wearable device data are highly personalized, the choice of optimal bin size may also need to be personalized depending on the person’s active hours.

## 5. Conclusions

Data from wearable devices could be a source of many missing values due to various reasons. It is not possible to always neglect the entire data for such reasons. This has driven the employment of several imputation methods based on statistical and machine learning approaches. In this study, we performed imputation using five well-known and successful imputation methods to show how using the bins of data for imputation could lower RMSE values. The EM imputation method was found to be the most successful method, with a ‘success’ rate of over 80%, which was followed by the II, SI, and kNN methods. RF was the only imputation method that did not show any significant changes in the RMSE values and remained ineffective throughout all the bin sizes. It was also found that the smallest bin size was the optimal bin size in most cases. However, the current study has some limitations. The current proposal may not be helpful if the user has highly varied activities over the course of a day. A study with more volunteers, including both healthy individuals and those suffering from different ailments, will improve our understanding.

## Data Availability

Data can be made available upon request to authors.

## Supplementary Materials

The following supporting information can be downloaded at https://www.mdpi.com/article/10.3390/s23031454/s1. Figure S1: Imputation of 1 h of missing data for volunteer V2. Variations in RMSE when data with different bin sizes were used for imputing missing data of ‘inactive’ period of 3–4 am using (a) EM, (b) II, (c) kNN, (d) RF, and (e) SI methods. Variations in RMSE when data with different bin sizes were used for imputing missing data of ‘active’ period of 3–4 pm using (f) EM, (g) II, (h) kNN, (i) RF, and (j) SI methods. Figure S2: Imputation of 15 min of missing data for volunteer V2. Variations in RMSE when data with different bin sizes were used for imputing missing data of ‘inactive’ period of 3–3:15 am using (a) EM, (b) II, (c) kNN, (d) RF, and (e) SI methods. Variations in RMSE when data with different bin sizes were used for imputing missing data of ‘active’ period of 3–3:15 pm using (f) EM, (g) II, (h) kNN, (i) RF, and (j) SI methods. Figure S3: Imputation of 1 h of missing data for volunteer V3. Variations in RMSE when data with different bin sizes were used for imputing missing data of ‘inactive’ period of 3–4 am using (a) EM, (b) II, (c) kNN, (d) RF, and (e) SI methods. Variations in RMSE when data with different bin sizes were used for imputing missing data of ‘active’ period of 3–4 pm using (f) EM, (g) II, (h) kNN, (i) RF, and (j) SI methods. Figure S4: Imputation of 15 min of missing data for volunteer V3. Variations in RMSE when data with different bin sizes were used for imputing missing data of ‘inactive’ period of 3–3:15 am using (a) EM, (b) II, (c) kNN, (d) RF, and (e) SI methods. Variations in RMSE when data with different bin sizes were used for imputing missing data of ‘active’ period of 3–3:15 pm using (f) EM, (g) II, (h) kNN, (i) RF, and (j) SI methods. Figure S5: Imputation of 1 h of missing data for volunteer V4. Variations in RMSE when data with different bin sizes were used for imputing missing data of ‘inactive’ period of 3–4 am using (a) EM, (b) II, (c) kNN, (d) RF, and (e) SI methods. Variations in RMSE when data with different bin sizes were used for imputing missing data of ‘active’ period of 3–4 pm using (f) EM, (g) II, (h) kNN, (i) RF, and (j) SI methods. Figure S6: Imputation of 15 min of missing data for volunteer V4. Variations in RMSE when data with different bin sizes were used for imputing missing data of ‘inactive’ period of 3–3:15 am using (a) EM, (b) II, (c) kNN, (d) RF, and (e) SI methods. Variations in RMSE when data with different bin sizes were used for imputing missing data of ‘active’ period of 3–3:15 pm using (f) EM, (g) II, (h) kNN, (i) RF, and (j) SI methods.
