# Supplementary material for: Binned Data Provide Better Imputation of Missing Time Series Data from Wearables
Source: Sensors (Basel). 2023 Jan 28;23(3):1454. doi: 10.3390/s23031454 (PMC9919790; doi:10.3390/s23031454)
Supplement: Supplementary file 1 [file sensors-23-01454-s001.zip › sensors-2113120-supplementary.pdf]

Supplementary Materials

Binned Data Provide Better Imputation of Missing Time Series Data from Wearables

Shweta Chakrabarti [1], Nupur Biswas [1,*], Khushi Karnani [2], Vijay Padul [1], Lawrence D. Jones [3], Santosh Kesari [4] and Shashaanka Ashili [3]

[1] Rhenix Lifesciences, Hyderabad 500038, India
[2] Department of BioSciences and BioEngineering, Indian Institute of Technology, Guwahati 781039, India
[3] CureScience, 5820 Oberlin Dr, 202, San Diego, CA 92121, USA
[4] Department of Translational Neurosciences, Pacific Neuroscience Institute and Saint John's Cancer Institute at Providence Saint John's Health Center, Santa Monica, CA 90404, USA
* Correspondence: nupur@rhenix.org or nupurbiswas@gmail.com

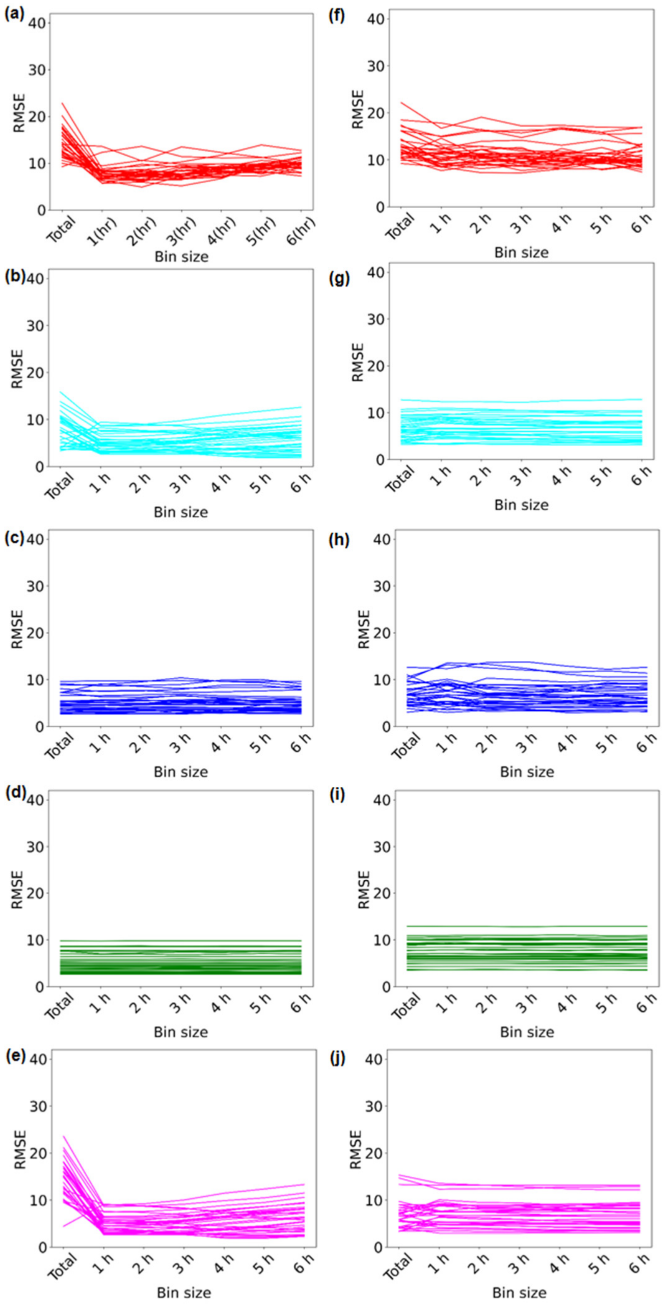

Figure S1: Imputation for 1 h missing data for volunteer V2. Variation in RMSE when data of different bin sizes was used for imputing missing data of 'inactive' period of 3–4 a.m. using (**a**) EM, (**b**) II, (**c**) kNN, (**d**) RF, and (**e**) SI methods respectively. Variation in RMSE when data of dif-ferent bin sizes was used for imputing missing data of 'active' period of 3–4 p.m. using (**f**) EM, (**g**) II, (**h**) kNN, (**i**) RF, and (**j**) SI methods respectively.

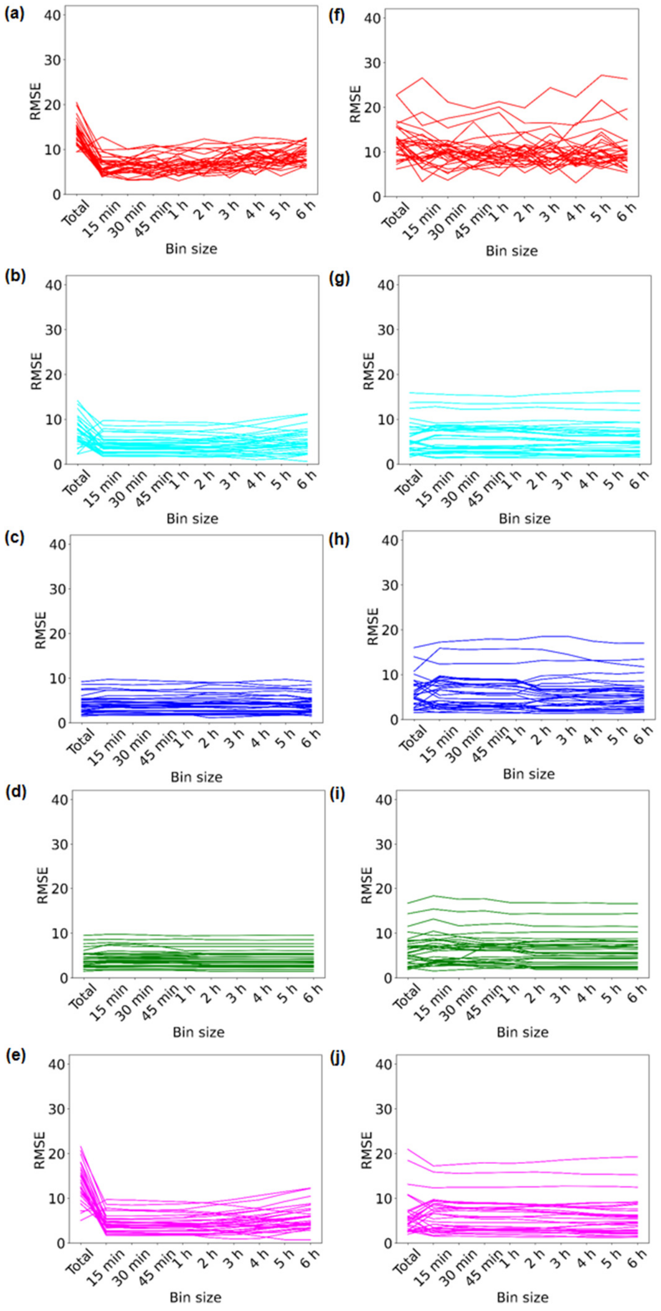

Figure S2: Imputation for 15 min missing data for volunteer V2. Variation in RMSE when data of different bin sizes was used for imputing missing data of 'inactive' period of 3–3:15 a.m. using (**a**) EM, (**b**) II, (**c**) kNN, (**d**) RF, and (**e**) SI methods respectively. Variation in RMSE when data of different bin sizes was used for imputing missing data of 'active' period of 3–3:15 p.m. using (**f**) EM, (**g**) II, (**h**) kNN, (**i**) RF, and (**j**) SI methods respectively.

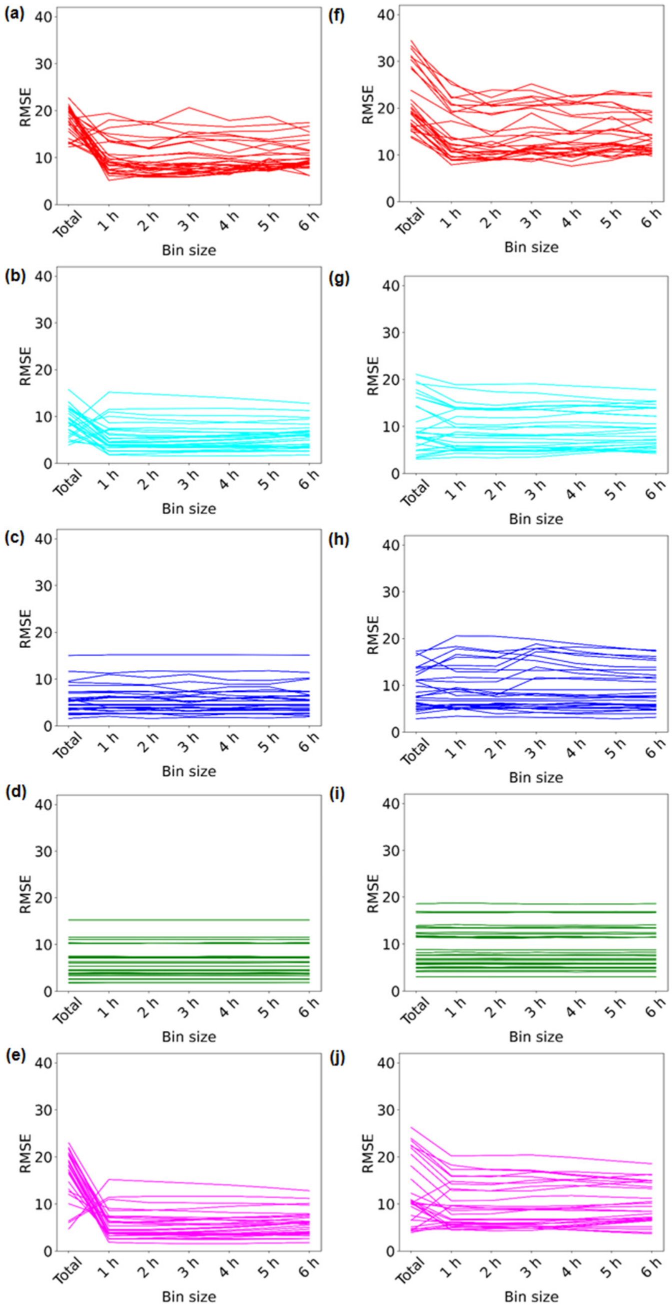

Figure S3: Imputation for 1 h missing data for volunteer V3. Variation in RMSE when data of different bin sizes was used for imputing missing data of ‘inactive’ period of 3–4 a.m. using (**a**) EM, (**b**) II, (**c**) kNN, (**d**) RF, and (**e**) SI methods respectively. Variation in RMSE when data of dif-ferent bin sizes was used for imputing missing data of ‘active’ period of 3–4 p.m. using (**f**) EM, (**g**) II, (**h**) kNN, (**i**) RF, and (**j**) SI methods respectively.

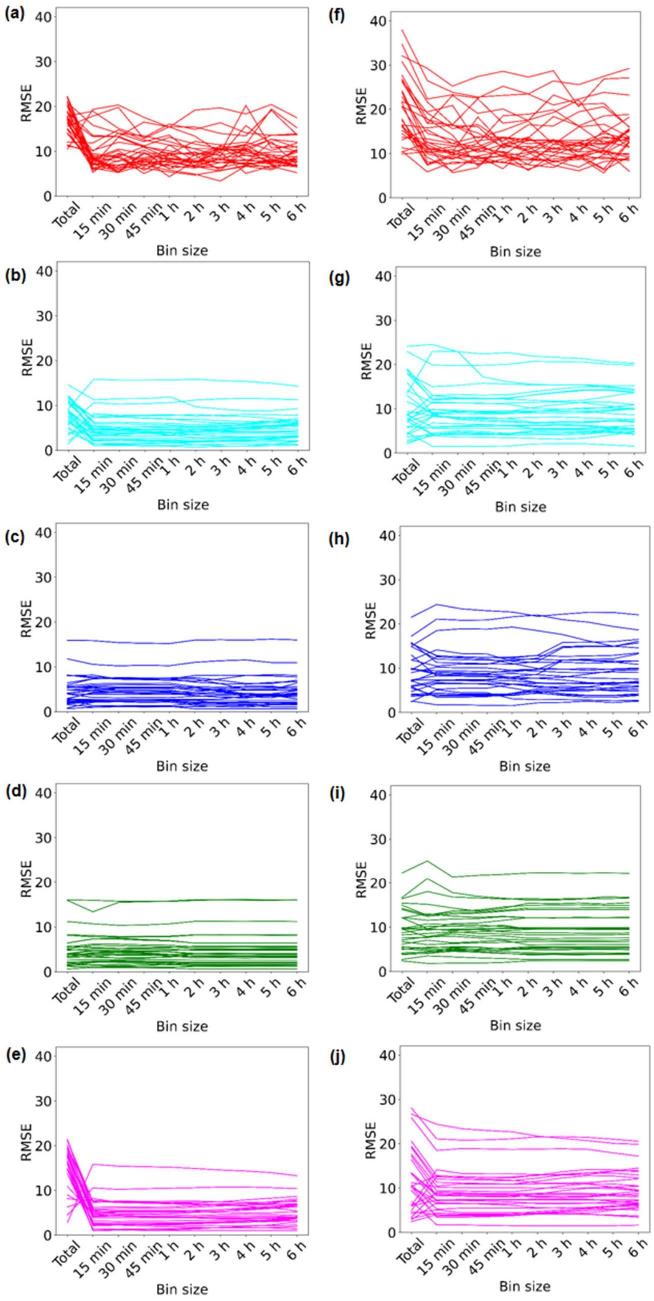

Figure S4: Imputation for 15 min missing data for volunteer V3. Variation in RMSE when data of different bin sizes was used for imputing missing data of 'inactive' period of 3–3:15 a.m. using (**a**) EM, (**b**) II, (**c**) kNN, (**d**) RF, and (**e**) SI methods respectively. Variation in RMSE when data of different bin sizes was used for imputing missing data of 'active' period of 3–3:15 p.m. using (**f**) EM, (**g**) II, (**h**) kNN, (**i**) RF, and (**j**) SI methods respectively.

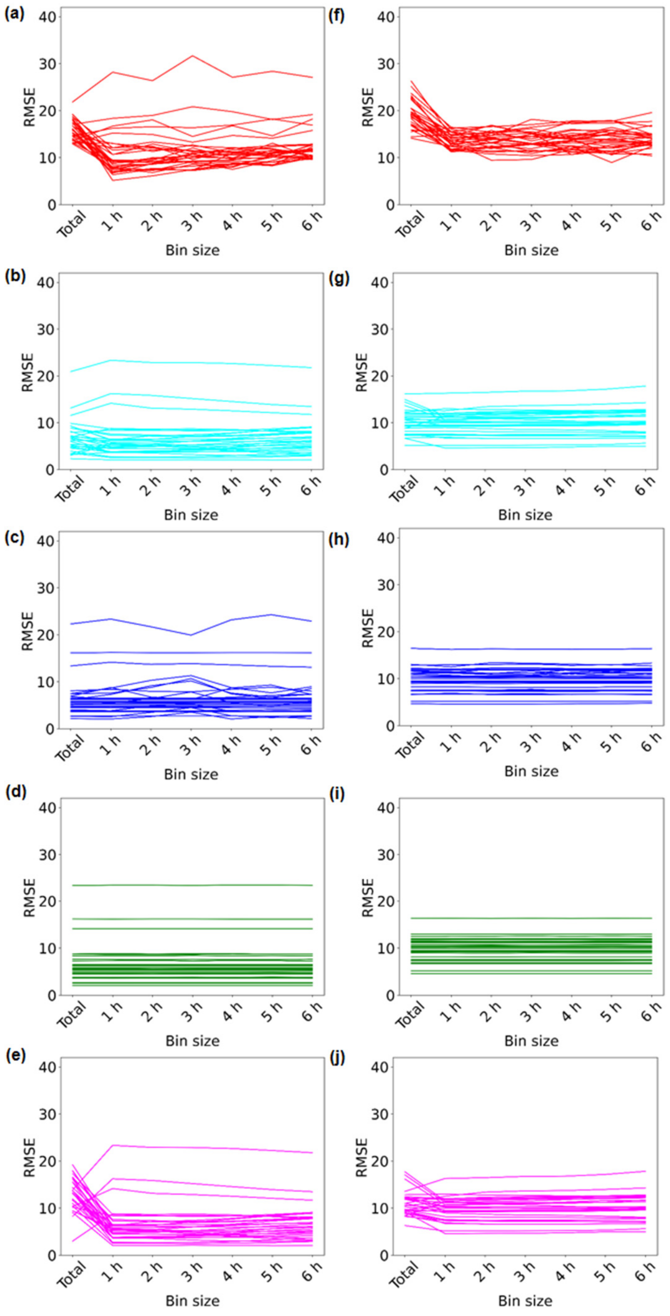

Figure S5: Imputation for 1 h missing data for volunteer V4. Variation in RMSE when data of different bin sizes was used for imputing missing data of 'inactive' period of 3–4 a.m. using (**a**) EM, (**b**) II, (**c**) kNN, (**d**) RF, and (**e**) SI methods respectively. Variation in RMSE when data of dif-ferent bin sizes was used for imputing missing data of 'active' period of 3–4 p.m. using (**f**) EM, (**g**) II, (**h**) kNN, (**i**) RF, and (**j**) SI methods respectively.

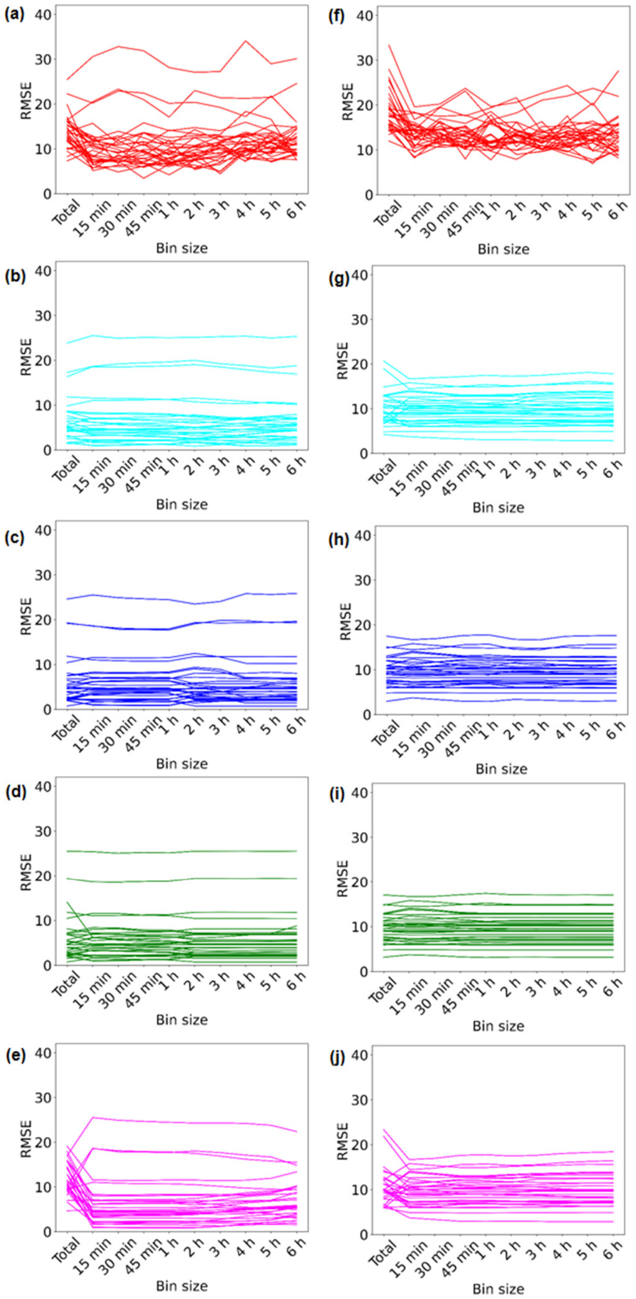

Figure S6: Imputation for 15 min missing data for volunteer V4. Variation in RMSE when data of different bin sizes was used for imputing missing data of ‘inactive’ period of 3–3:15 a.m. using (**a**) EM, (**b**) II, (**c**) kNN, (**d**) RF, and (**e**) SI methods respectively. Variation in RMSE when data of different bin sizes was used for imputing missing data of ‘active’ period of 3–3:15 p.m. using (**f**) EM, (**g**) II, (**h**) kNN, (**i**) RF, and (**j**) SI methods respectively.
